# Kirkwood-Buff Integrals Using Molecular Simulation: Estimation of Surface Effects

**DOI:** 10.3390/nano10040771

**Published:** 2020-04-16

**Authors:** Noura Dawass, Peter Krüger, Sondre K. Schnell, Othonas A. Moultos, Ioannis G. Economou, Thijs J. H. Vlugt, Jean-Marc Simon

**Affiliations:** 1 Engineering Thermodynamics, Process & Energy Department, Faculty of Mechanical, Maritime and Materials Engineering, Delft University of Technology, Leeghwaterstraat 39, 2628CB Delft, The Netherlandst.j.h.vlugt@tudelft.nl (T.J.H.V.); 2 Graduate School of Engineering and Molecular Chirality Research Center, Chiba University, Chiba 263-8522, Japan; 3 Department of Materials Science and Engineering, NTNU, N-7491 Trondheim, Norway; 4 National Center for Scientific Research Demokritos, Institute of Nanoscience and Nanotechnology, Molecular Thermodynamics and Modelling of Materials Laboratory, GR 153 10 Aghia Paraskevi Attikis, Greece; 5 Chemical Engineering Program, Texas A&M University at Qatar, Education City, Doha PO Box 23874, Qatar; 6 ICB, UMR 6303 CNRS-Université de Bourgogne, F-21078 Dijon, France

**Keywords:** nanothermodynamics, Kirkwood-Buff integrals, surface effects, molecular dynamics

## Abstract

Kirkwood-Buff (KB) integrals provide a connection between microscopic properties and thermodynamic properties of multicomponent fluids. The estimation of KB integrals using molecular simulations of finite systems requires accounting for finite size effects. In the small system method, properties of finite subvolumes with different sizes embedded in a larger volume can be used to extrapolate to macroscopic thermodynamic properties. KB integrals computed from small subvolumes scale with the inverse size of the system. This scaling was used to find KB integrals in the thermodynamic limit. To reduce numerical inaccuracies that arise from this extrapolation, alternative approaches were considered in this work. Three methods for computing KB integrals in the thermodynamic limit from information of radial distribution functions (RDFs) of finite systems were compared. These methods allowed for the computation of surface effects. KB integrals and surface terms in the thermodynamic limit were computed for Lennard–Jones (LJ) and Weeks–Chandler–Andersen (WCA) fluids. It was found that all three methods converge to the same value. The main differentiating factor was the speed of convergence with system size *L*. The method that required the smallest size was the one which exploited the scaling of the finite volume KB integral multiplied by *L*. The relationship between KB integrals and surface effects was studied for a range of densities.

## 1. Introduction

Using knowledge of the molecular structure of liquids to predict their macroscopic behavior is important for several applications [1,2,3,4,5]. One of the most rigorous solution theories is the Kirkwood–Buff (KB) theory, where a sound connection between macroscopic and microscopic properties for isotropic multicomponent fluids is established [6,7]. Kirkwood and Buff derived a relation between several thermodynamic properties and integrals of radial distribution functions (RDFs) over infinite and open volumes $G_{\alpha \beta }^{\infty }$ in the grand-canonical ensemble [6]:

(1) $$G_{\alpha \beta }^{\infty }=\int_{0}^{\infty }\left[g_{\alpha \beta }^{\infty }\left(r\right)-1\right]4\pi r^{2}dr$$

where *r* is the particle distance, and $g_{\alpha \beta }^{\infty }\left(r\right)$ is the RDF, of the infinitely large system, for species α and β. KB integrals can also be expressed in terms of density fluctuations in open systems [6,7,8,9]. While KB integrals were derived for open and infinite systems, many studies use molecular simulation to estimate KB integrals, where only finite systems can be studied. In Reference [10], a review of the methods available in literature for computing KB integrals from molecular simulations is presented.

To accurately estimate $G_{\alpha \beta }^{\infty }$, it is possible to use KB integrals of finite and open subvolumes *V* embedded in larger reservoirs. In this way, the grand-canonical ensemble, in which KB integrals in the thermodynamic limit were derived, is mimicked. This approach is referred to as the small system method (SSM) [5,11,12]. According to the SSM, properties of small subvolumes, that can be of the order of a few molecular diameters, are treated in terms of thermodynamics of small systems rather than classical thermodynamics. According to Hill’s thermodynamics of small systems, properties of open embedded subvolumes scale with the inverse size of the subvolumes [13,14]. This also applies to KB integrals of finite subvolumes, $G_{\alpha \beta }^{V}$ [10,12]. For a specific system, $G_{\alpha \beta }^{V}$ computed with subvolumes of different sizes, scales linearly with the inverse size of the subvolume [10,12,15,16]. For spherical subvolumes *V* inside a simulation box, we have

(2) $$G_{\alpha \beta }^{V}\left(L\right)=G_{\alpha \beta }^{\infty }+\frac{F^{\infty }}{L}$$

where L=6V/A is the characteristic length of the subvolume *V* with surface area A. For a sphere, *L* is the diameter (L=2R). In Equation (2), $F^{\infty }$ is related to surface effects of the subvolume. Using Hill’s formulation of small-system thermodynamics [14], it was shown that properties of small systems can be written in terms of volume and surface contributions [17]. In Reference [17], Hill’s thermodynamics were applied to several properties, including pressure. From the volume contribution of pressure, the homogeneous pressure is obtained, while the Gibbs surface relation was obtained from the surface contribution [17]. This last contribution is proportional to the surface tension. In the case of KB integrals, the surface term, or contribution, $F^{\infty }$, can also be defined from Gibbs surface equation [17]. From a microscopic point of view, it originates from interactions between molecules inside the subvolume and molecules across the boundary of the subvolume [12,15]. These surface effects vanish in the thermodynamic limit, but for systems used in molecular dynamics (MD) simulations these effects cannot be neglected [18]. As a result, the quantitative and qualitative study of surface contributions is essential for estimating $G_{\alpha \beta }^{\infty }$ from integrals of finite subvolumes $G_{\alpha \beta }^{V}$.

KB integrals of finite and open subvolumes $G_{\alpha \beta }^{V}$ are defined in terms of fluctuations in the number of particles, which relate to double integrals of RDFs over the subvolume *V* [12],

(3) $$G_{\alpha \beta }^{V}\equiv \frac{1}{V}\int_{V}\int_{V}\left[g_{\alpha \beta }\left(r_{12}\right)-1\right]dr_{1}dr_{2}\equiv V\frac{\langle N_{\alpha }N_{\beta }\rangle -\langle N_{\alpha }\rangle \langle N_{\beta }\rangle }{\langle N_{\alpha }\rangle \langle N_{\beta }\rangle }-\frac{V\delta_{\alpha \beta }}{\langle N_{\beta }\rangle }$$

where $N_{\alpha }$ and $N_{\beta }$ are the number of molecules of type α and β, in volume *V*. The brackets 〈⋯〉 denote an ensemble average in an open system. Equation (3) is applicable to isotropic molecular fluids where the orientations of molecules are already integrated out. While it is possible to compute KB integrals $G_{\alpha \beta }^{V}$ from fluctuations in the number of particles (i.e., the right hand side of Equation (3)), it is more practical to use RDFs. RDFs are readily computed by most molecular simulation software packages. The double integrals in the left hand side of Equation (3) can be transformed to a single integral using a weight function w(r,L) [12],

(4) $$G_{\alpha \beta }^{V}=\int_{0}^{L}\left[g_{\alpha \beta }\left(r\right)-1\right]w\left(r,L\right)dr$$

The function w(r,L) depends on the geometry of *V*. For spherical and cubic subvolumes, theoretically derived functions are available in References [12,16], respectively. It is possible to numerically obtain the function w(r,L) for an arbitrary shape as shown in Reference [19]. In this work, spherical subvolumes will be used, for which,

(5) $$w\left(r,L\right)=4\pi r^{2}\left(1-\frac{3x}{2}+\frac{x^{3}}{2}\right)$$

where *x* is the dimensionless distance r/L [12,15,16]. The scaling of finite integrals $G^{V}$ with the size of the subvolumes *L* is used to compute KB integrals in the thermodynamic limit $G^{\infty }$ (for convenience, indicies α and β will be dropped from this point onwards). Specifically, $G^{\infty }$ is computed from extrapolating the linear part of the scaling of $G^{V}$ with 1/L to the limit 1/L→0 [12,15,17]. A disadvantage of this approach is that a linear regime is not always easily identified [15].

To avoid extrapolating $G^{V}$, Krüger and Vlugt [16] proposed a direct estimation of KB integrals in the thermodynamic limit:

(6) $$G^{\infty }\approx G_{k}\left(L\right)=\int_{0}^{L}\left[g(r)-1\right]u_{k}\left(r\right)dr$$

The accuracy of the estimation depends on the function $u_{k}\left(r\right)$ [20]. Krüger and Vlugt [16] considered three different estimations and found that integrals computed using the function $u_{2}\left(r\right)$ provided the best estimation of $G^{\infty }$,

(7) $$u_{2}\left(r\right)=4\pi r^{2}\left(1-\frac{23}{8}x^{3}+\frac{3}{4}x^{4}+\frac{9}{8}x^{5}\right)$$

KB integrals computed using Equations (6) and (7) will be denoted by $G_{2}$. To derive the expression for $G_{2}$, the starting point was the scaling of KB integrals with 1/L. First, an explicit estimation of $F^{\infty }$ in Equation (2) was derived. In the work of Krüger and Vlugt [16], $F^{\infty }$ has the following form

(8) $$F^{\infty }=\int_{0}^{\infty }\left[g\left(r\right)-1\right]4\pi r^{2}\left(-\frac{3}{2}r\right)dr$$

It is important to note that the structure of Equation (8) is similar to KB integrals in the thermodynamic limit (Equation (1)). So, analogous to Equation (2), $F^{V}$ can be defined as,

(9) $$F^{V}\left(L\right)=F^{\infty }+\frac{C}{L}$$

where *C* is a constant. For finite systems, $F^{V}$ can be computed using

(10) $$F^{V}\approx \int_{0}^{L}\left[g\left(r\right)-1\right]\left(-\frac{3}{2}r\right)w\left(r,L\right)dr$$

where the function w(r,L) is given in Equation (5). The similarity between the expression for KB integrals (Equation (4)) and surface term (Equation (8)) in the thermodynamic limit allows for deriving an estimation for surface effects as in Equation (6). Using Equation (6), and Equation (8) an explicit expression for surface effects in the thermodynamic limit, denoted here by $F_{2}^{\infty }$, is obtained from

(11) $$F_{2}^{\infty }\approx \int_{0}^{L}\left[g\left(r\right)-1\right]\left(-\frac{3}{2}r\right)u_{2}\left(r\right)dr$$

with $u_{2}\left(r\right)$ in Equation (7).

An alternative method to extrapolate KB integrals $G^{V}$ to the thermodynamic limit is to use the scaling of $LG^{V}$ with *L*, rather than the scaling of $G^{V}$ with 1/L. The scaling of $G^{V}$ in Equation (2) can be rewritten as

(12) $$LG^{V}\left(L\right)=G^{\infty }L+F^{\infty }$$

By fitting the linear part of the scaling of $LG^{V}$ with *L*, it is possible to obtain $G^{\infty }$ and $F^{\infty }$. Finding the slope and intercepts of a straight line is easier than extrapolating the linear regime of the scaling of $G^{V}$ with 1/L. Another advantage of this approach is that an estimation of the surface effects is automatically computed. This estimation can be compared to other available methods for computing $F^{\infty }$. So far, it is shown that three methods are available for estimating $G^{\infty }$ from integrals of finite subvolumes:

1. Using the scaling of $G^{V}$ (Equation (4)) with 1/L. To estimate $G^{\infty }$, the linear regime of the scaling is extrapolated to the limit 1/L→0.
2. Using the direct extrapolation formula $G_{2}$ (Equation (6)) combined with the function $u_{2}\left(r\right)$ (Equation (7)). This will converge to $G^{\infty }$ for large *L*.
3. Computing $G^{\infty }$ from fitting the linear regime of the scaling of $LG^{V}$ with *L* (Equation (12)). The values of the integrals $G^{V}$ are computed using Equation (4).

To simplify the estimation of KB integrals, it would be useful to evaluate the performance of these methods in terms of accuracy and practicality. Similarly, different methods are available to compute the surface term in the thermodynamic limit $F^{\infty }$:

1. Using the expression in Equation (11).
2. From the scaling of $LF^{V}$ with *L* (Equation (9)). $F^{V}$ is computed using Equation (10). The value of $F^{\infty }$ is obtained from the slope of the scaling; as $LF^{V}\left(L\right)=F^{\infty }L+C$, in which *C* is a constant.
3. From the scaling of $LG^{V}$ with *L* (Equation (9)). The value of $F^{\infty }$ is obtained from the intercept of the scaling.

The objective of this work is to test the estimation of KB integrals $G^{\infty }$ and the surface effects $F^{\infty }$ using the approaches discussed earlier. For both $G^{\infty }$ and $F^{\infty }$, the effect of the size of the system is studied. These effects are investigated for both Lennard–Jones (LJ) and Weeks–Chandler–Andersen (WCA) fluids [21] at different densities. Finally, this work aims at quantifying the contributions of the surface term when computing KB integrals of LJ fluid at various densities.

This paper is organized as follows: In Section 2, the methods used to compute RDFs, KB integrals, and the surface term of KB integrals of LJ and WCA fluids are presented. Section 2 includes the details of the MD simulations. In Section 3, the results are presented, which include KB integrals and the surface term for WCA and LJ systems at different sizes and densities. Section 4 summarises the main findings of this work.

## 2. Methods

RDFs of systems of particles interacting via the LJ potential are computed using MD simulations in the NVT ensemble. Systems with different densities and number of particles are studied. Also, systems of particles interacting via the Weeks–Chandler–Andersen (WCA) potential [22], where only the repulsive part of the LJ potential is included, are considered. The common approach of particles counting was implemented to compute RDFs. While this is not carried out in this work, it is possible to investigate other methods. It would be interesting to see if force-based computations of RDFs improve the convergence of computed KB integrals [23,24,25]. For each system, the computed RDF is used to compute KB integrals $G^{\infty }$ and the surface term $F^{\infty }$ in the thermodynamic limit. For both quantities, the methods discussed in Section 1 are used. In this section, the numerical details of computing RDFs and the required integrals are briefly discussed.

According to Kirkwood–Buff theory, KB integrals are defined for open and infinite systems [6]. When computing KB integrals using molecular simulations of closed systems, it is essential to correct RDFs for finite-size effects [10,12,15]. Recently, a number of corrections for the RDFs have been proposed [12,26,27]. In Reference [15] it was demonstrated that the accuracy of computing KB integrals improves when the Ganguly and van der Vegt correction [26] is applied. Applying the Ganguly and van der Vegt correction results in RDFs that are consistent with the physical behavior of fluids. For example, Equation (13) converges to g(r)=1 for a single-component ideal gas, which is the correct value in the thermodynamic limit. The Ganguly and van der Vegt [26] correction is based on the excess (or depletion) of the density of the system beyond a distance *L* from a central molecule α. The corrected RDF is

(13) $$g_{\alpha \beta }^{\mathrm{vdV}}\left(r\right)=g_{\alpha \beta }\left(r\right)\frac{N_{\beta }\left(1-\frac{V}{V_{\mathrm{box}}}\right)}{N_{\beta }\left(1-\frac{V}{V_{\mathrm{box}}}\right)-\Delta N_{\alpha \beta }\left(r\right)-\delta_{\alpha \beta }}$$

$g_{\alpha \beta }\left(r\right)$ is obtained from a simulation in a finite system with total volume $V_{\mathrm{box}}$. $\Delta N_{\alpha \beta }\left(r\right)$ is the excess number of particles of type β in a sphere of radius *r* around a particle of type α, which is computed by

(14) $$\Delta N_{\alpha \beta }\left(r\right)=\int_{0}^{r}dr^{\prime }4\pi r^{\prime 2}\rho_{\beta }\left[g_{\alpha \beta }\left(r^{\prime }\right)-1\right]$$

For all systems studied in this work, RDFs are corrected using the Ganguly and van der Vegt corrections. The corrected RDFs are numerically integrated to obtain $G^{V}$, $G_{2}$, $F^{V}$, and $F_{2}^{\infty }$. Once these quantities are obtained, various methods are implemented to estimate KB integrals $G^{\infty }$ and the surface terms $F^{\infty }$ in the thermodynamic limit. Table 1 provides the relations and description of the methods considered to estimate $G^{\infty }$. Similarly, Table 2 presents information regarding the methods used to estimate $F^{\infty }$.

### Simulation Details

RDFs of LJ and WCA fluids were computed using MD simulations and then used to estimate KB integrals and surface effects. The simulations were carried out using an in-house FORTRAN code. All RDFs were computed from simulations in the NVT ensemble. The systems were simulated at a dimensionless temperature T=2, dimensionless densities ρ ranging from 0.2 to 0.8 and using number of particles *N* equals to 100, 500, 1000, 5000, 10,000, 30,000, and 50,000. For each size, the length of the simulation box *L* was set according to the required density.

All MD simulations started from a randomly generated configuration for which an energy minimization was used to eliminate particle overlaps. A sufficient number of time steps was used to initialize the system. After initialization, RDFs were sampled every 100 time steps. For both initialization and production, a dimensionless time step equal to 0.001 was used. The simulation length was chosen depending on the size of the system and the available computational resources. For instance, for systems with N=100, 1 ×$10^{9}$ production time steps were carried out, while for the maximum size *N* = 50,000, 7 ×$10^{5}$ steps were used. Multiple independent simulations were performed for each point (ρ, *N*). The resulting RDFs were then averaged and used to compute $G^{\infty }$ and $F^{\infty }$. At high densities (ρ>0.4), RDFs from at least 10 runs are used. At lower densities, at least 20 runs are performed to enhance statistics.

## 3. Results

### 3.1. Estimation of KB Integrals

KB integrals in the thermodynamic limit $G^{\infty }$ are obtained using the three different approaches discussed earlier. To compare the estimation methods, WCA systems were studied while fixing temperature and density. These parameters define the thermodynamic state of the system. Values of KB integrals, computed using different methods, for other densities for LJ and WCA fluids are provided in the Supporting Information (SI). After comparing estimation methods of KB integrals, the relation between density of the system and KB integrals for LJ and WCA system is discussd.

**Figure 1:**
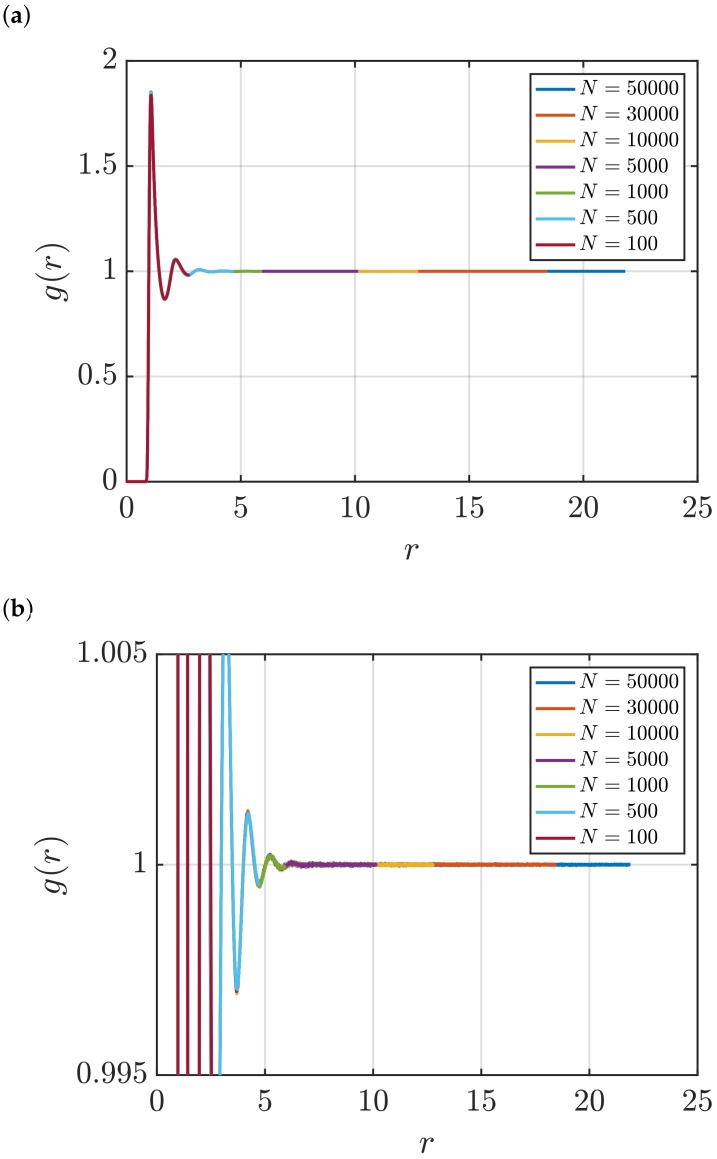
(**a**) RDFs for systems of different sizes of a Weeks–Chandler–Andersen (WCA) fluid at *T* = 2 and *ρ* = 0.6 (dimensionless units). Molecular dynamics (MD) simulations in the *NVT* ensemble were used to compute *g*(*r*), and the Ganguly and van der Vegt correction [26] was applied (Equation (13)). (**b**) A zoom in of the plots in Figure (**a**) is shown.

**Figure 2:**
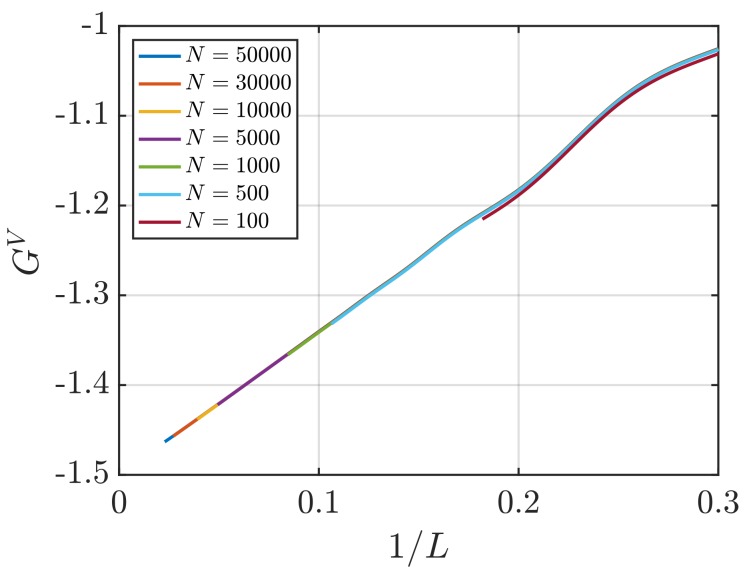
KB integrals of finite spherical subvolumes $G^{V}$ (Equation (4)) vs. 1/L (*L* is the diameter) for the WCA fluid at T=2 and ρ=0.6 (dimensionless units). The values of $G^{V}$ are computed for systems with varying number of molecules *N*. The used RDFs are provided in Figure 1.

**Figure 3:**
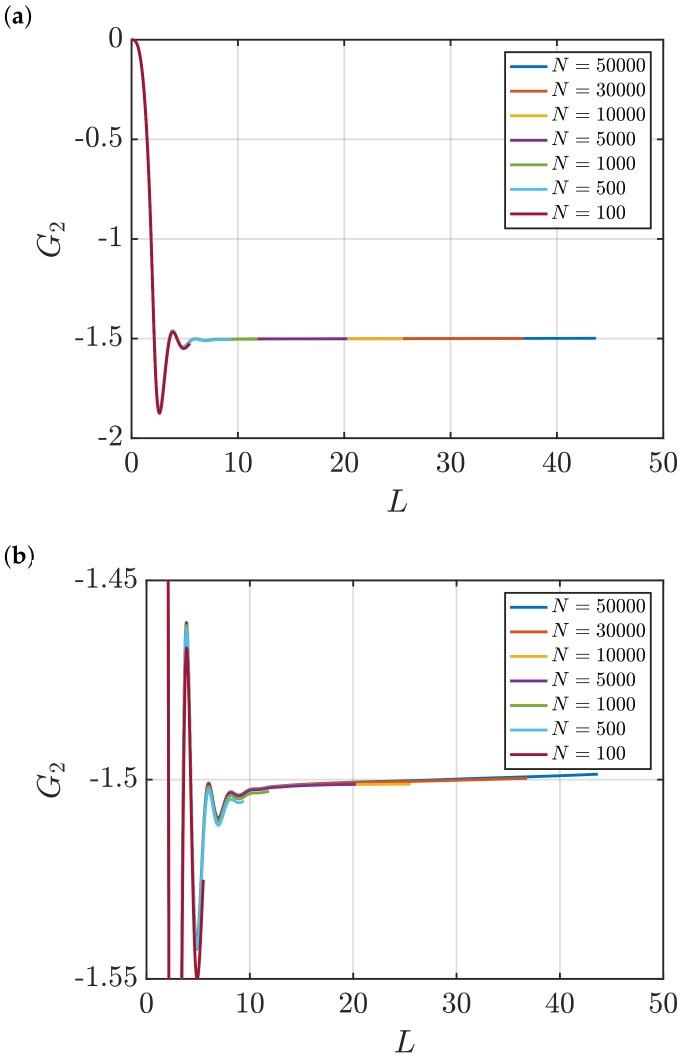
(**a**) Estimation of KB integrals in the thermodynamic limit, *G*_2_ (Equations (6) and (7)) vs. *L* for the WCA fluid at *T* = 2 and *ρ* = 0.6 (dimensionless units). The values of *G*_2_ are computed for systems with varying number of molecules *N*. The used RDFs are provided in Figure 1. (**b**) A zoom in of the plots in Figure (**a**) is shown.

**Figure 4:**
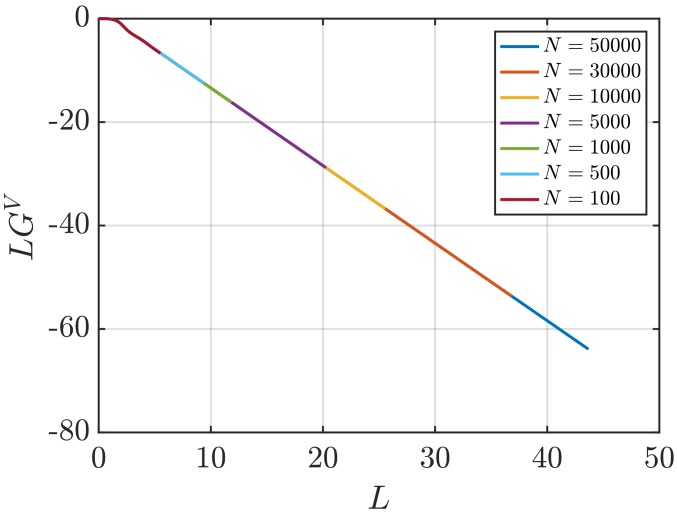
KB integrals of finite subvolumes multiplied by *L*, $LG^{V}$ (Equation (4)) vs. *L* for a WCA fluid at T=2 and ρ=0.6 (dimensionless units). The values of $G^{V}$ are computed for systems with varying number of molecules *N*. The used RDFs are provided in Figure 1.

Figure 1 shows RDFs for systems of different sizes of a WCA fluid at T=2 and ρ=0.6 (dimensionless units). Figure 1b shows that using small system sizes, specifically N=100 and N=500, results in RDFs with higher oscillations than large systems, where *N* equals to or larger than 1000. As will be shown later, this causes implications in the computation of $G^{\infty }$. In Figure 2, the scaling of KB integrals of finite subvolumes $G^{V}$ with 1/L is presented. For large systems, where N>500, a linear range is identified which can be extrapolated to the limit 1/L→0. Instead of computing $G^{V}$, KB integrals $G^{\infty }$ can be directly estimated from RDFs using Equations (6) and (7). Figure 3 shows the estimation of $G_{2}$ for systems with varying sizes. When plotted as a function of *L*, the values of the integrals $G_{2}$ show a plateau at a constant value which corresponds to $G^{\infty }$. However, Figure 3b shows that this is not true for all system sizes. In fact, the values of $G^{\infty }$ can be accurately estimated for systems with a minimum number of particles of 5000, which is larger than the minimum size required in the previous extrapolation method (Figure 2). The third method to find $G^{\infty }$ is to use the scaling of $LG^{V}$ with *L* (Equation (2)). Figure 4 shows that plotting the integrals of finite subvolumes as $LG^{V}$ vs. *L* results in a clear linear regime that is easily identified. The value of the slope of the fitted line corresponds to the value of $G^{\infty }$. For this method, systems with number of particles equal to or larger than 500 can already be used to compute $G^{\infty }$. In principle, all methods for estimating $G^{\infty }$ should result in the same answer in the thermodynamic limit. In Table 3, values of KB integrals $G^{\infty }$ obtained using the three methods studied in this work are listed. For KB integrals reported in Table 3, only uncertainties greater than 0.01% are shown. The values are obtained from systems with various sizes. For each size, a linear range was used to compute $G^{\infty }$. In Reference [15], guidelines were provided for selecting a range for the extrapolation of $G^{V}$ vs. 1/L. Essentially, the first few molecular diameters after r=0, and distances beyond L/2 should be avoided. Fitting lines of the scaling of $LG^{V}$ vs. *L* is more convenient. In general, fitting regions are chosen such that the coefficient of determination $R^{2}$ is equal to or very close to 1. The values of $G^{\infty }$ in Table 3 show that the three methods provide very similar estimations with statistical uncertainties below 0.1%. Moreover, results in Table 3 show that computing $G^{\infty }$ using the direct estimation $G_{2}$ require larger systems compared to the other methods. This was found to be true for other densities as well as for systems with LJ particles (see the SI). From studying other systems, it was found that the scaling of $LG^{V}$ with *L* is found to be the easiest method to apply.

**Figure 5:**
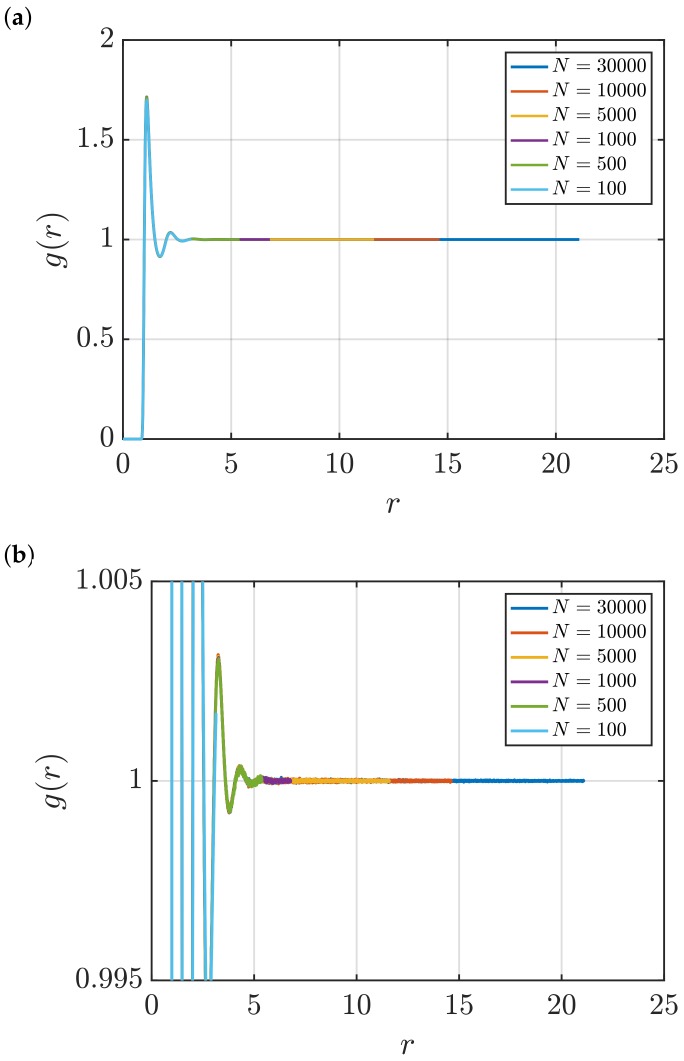
(**a**) RDFs for systems of different sizes of a LJ fluid at *T* = 2 and *ρ* = 0.4 (dimensionless units). MD simulations in the *NVT* ensemble were used to compute *g*(*r*), and the Ganguly and van der Vegt correction [26] was applied (Equation (13)). (**b**) A zoom in of the plots in Figure (**a**) is shown.

**Figure 6:**
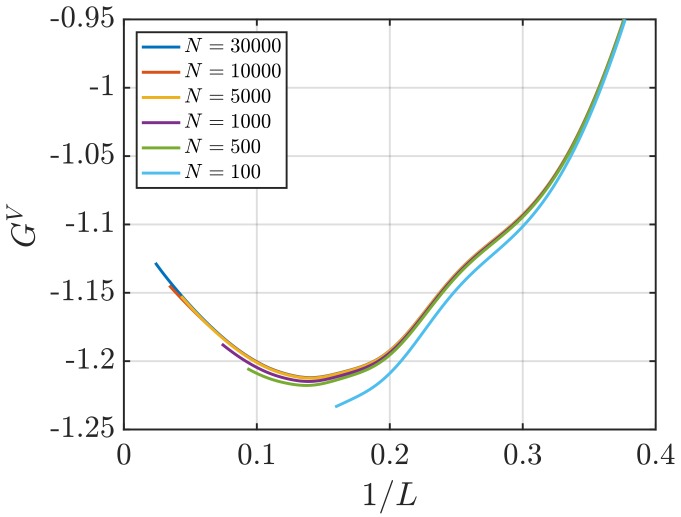
KB integrals of finite spherical subvolumes $G^{V}$ (Equation (4)) vs. 1/L (*L* is the diameter) for the LJ fluid at T=2 and ρ=0.4 (dimensionless units). The values of $G^{V}$ are computed for systems with varying number of molecules *N*. The used RDFs are provided in Figure 5.

**Figure 7:**
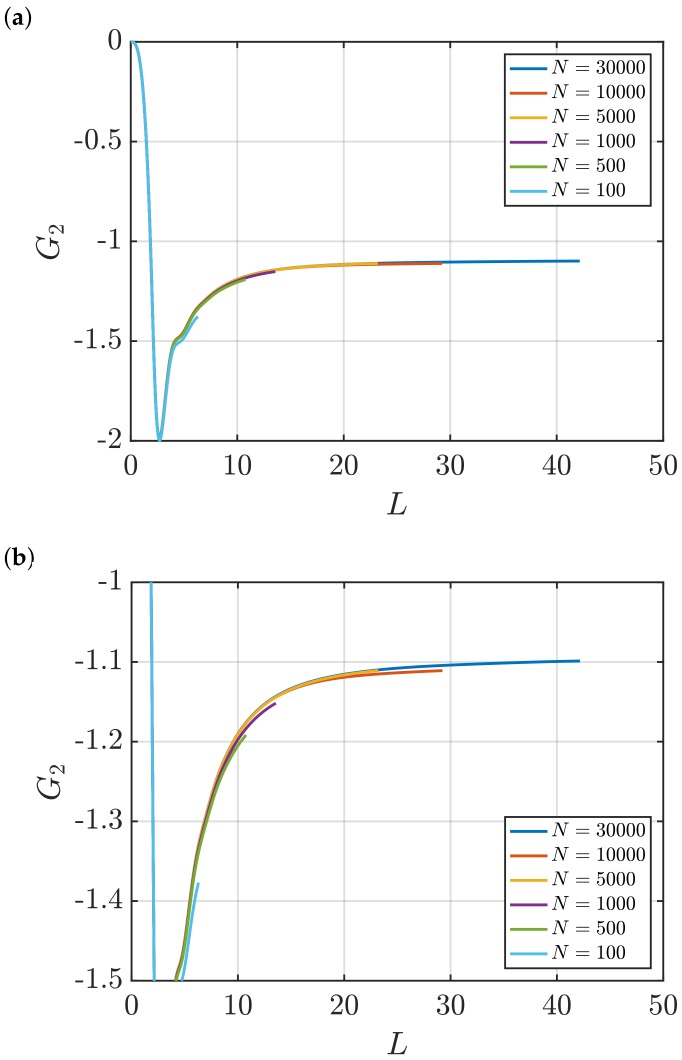
(**a**) Estimation of KB integrals in the thermodynamic limit, *G*_2_ (Equations (6) and (7)) vs. *L* for the LJ fluid at *T* = 2 and *ρ* = 0.4 (dimensionless units). The values of *G*_2_ are computed for systems with varying number of molecules *N*. The used RDFs are provided in Figure 5. (**b**) A zoom in of the plots in Figure (**a**) is shown.

**Figure 8:**
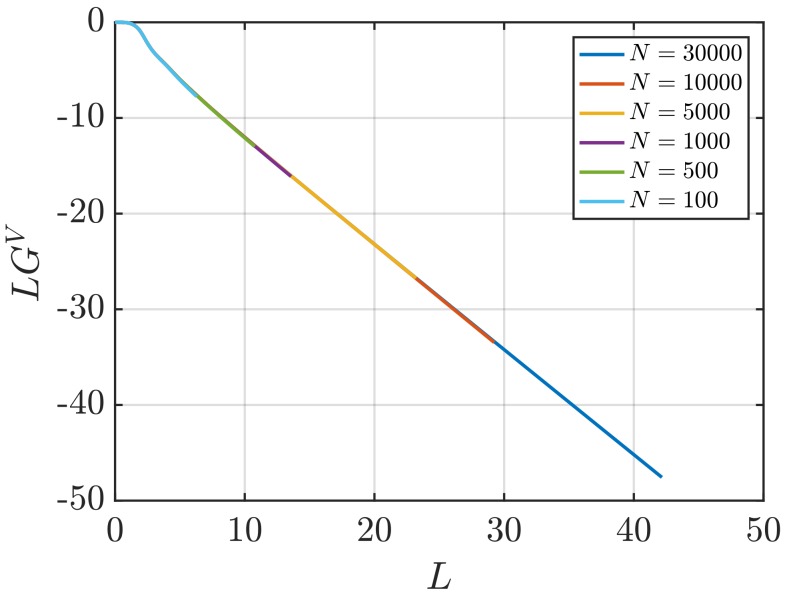
KB integrals of finite subvolumes multiplied by *L*, $LG^{V}$ (Equation (4)) vs. *L* for a LJ fluid at T=2 and ρ=0.4 (dimensionless units). The values of $G^{V}$ are computed for systems with varying number of molecules *N*. The used RDFs are provided in Figure 5.

The differences between the estimation methods can be further demonstrated by using a system of LJ particles at ρ=0.4, which is more difficult to sample compared to the previously studied system. In Figure 5, RDFs of systems of varying sizes of a LJ fluid at ρ=0.4 and T=2 are shown. In Table 4, KB integrals $G^{\infty }$ computed using the RDFs in Figure 5 are provided. The scaling of $G^{V}$ with 1/L is shown in Figure 6. For this method, a linear range is not obtained for all system sizes. Systems with at least N=1000 particles can be used to extrapolate to the thermodynamic limit. In Figure 7, KB integrals $G_{2}$ are plotted as a function of the size of the subvolume *L*. The figure shows that even larger systems are needed to find a reasonable estimate of $G^{\infty }$ using $G_{2}$. Figure 7b shows that a plateau is only achieved for large systems where *N* equals to or larger than 5000. For this system (LJ fluid at ρ=0.4), it is possible to use the scaling of $LG^{V}$ with *L* to compute KB integrals from small sizes. Figure 8 demonstrates that straight lines that are easily fitted are achieved when using the scaling of $LG^{V}$ with *L*, even for sizes where an estimation can not be made with the other two methods.

#### Effect of System Size and Density

**Figure 9:**
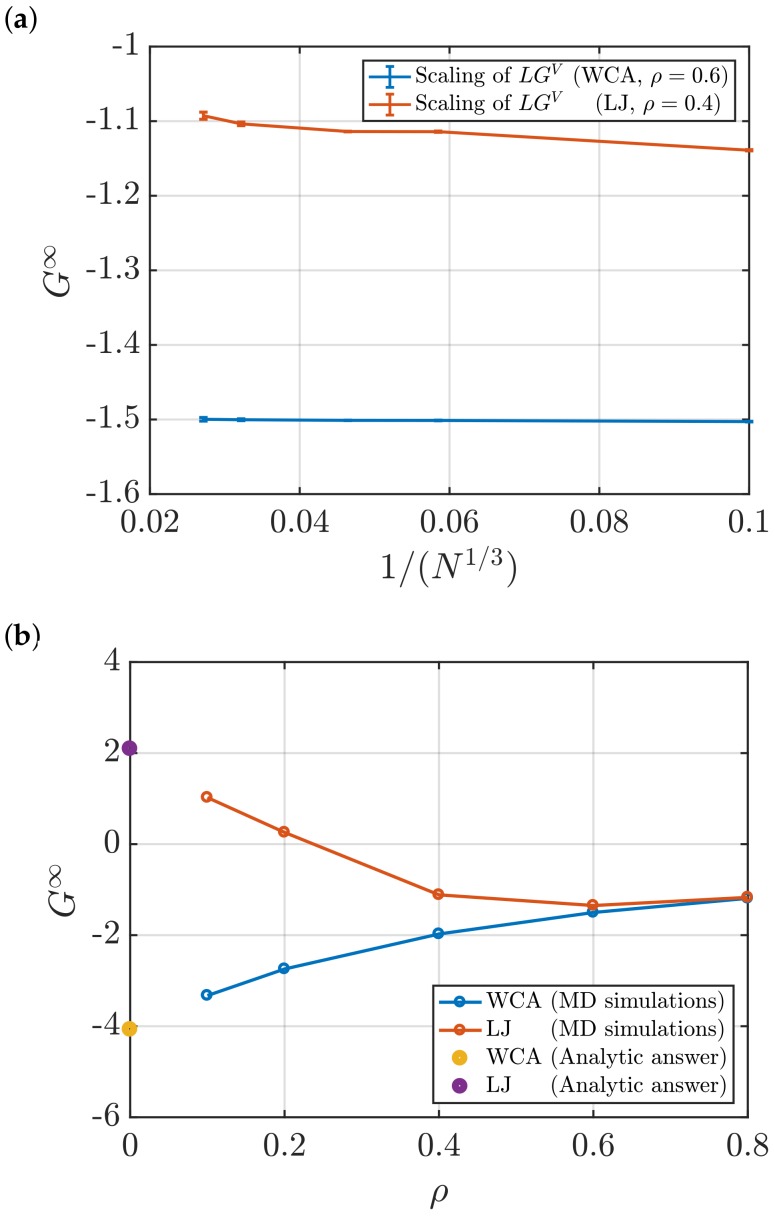
(**a**) KB integrals in the thermodynamic limit *G*^∞^ as a function of the size of the system for the WCA fluid at *ρ* = 0.6, and the LJ fluid at *ρ* = 0.4. Both fluids are simulated at *T* = 2 (dimensionless units). (**b**) *G*^∞^ as a function of dimensionless density *ρ* of LJ and WCA systems at *T* = 2. For all densities, the same number of particles is used, *N* = 10,000.

Figure 9a shows the effect of the size of the system on the values of $G^{\infty }$ computed using the scaling of $LG^{V}$ with *L*. The obtained values of $G^{\infty }$ are practically constant. For the LJ fluid, a weak decrease, roughly linear in $N^{-1/3}$, is observed. Figure 9a, Table 3 and Table 4 demonstrate that statistical uncertainties are small for systems with intermediate sizes (N=5000 and *N* = 10,000). Smaller systems do not provide a sufficient linear regime and very large systems require longer sampling. In Figure 9b, KB integrals at different densities are shown for the LJ and WCA fluids. To estimate $G^{\infty }$, the scaling of $LG^{V}$ with *L* was used. MD simulations were performed to study systems with dimensionless densities ranging from 0.1 to 0.8.

The behaviour of KB integrals in the limit ρ→0 can be checked by using the fact that in this limit, the RDF is known analytically, g(r)=exp[−βu(r)], where u(r) is the pair potential [28]. Figure 9b shows that for both interaction potentials, the values of $G^{\infty }$ computed using molecular simulation approach the correct value in the low density limit. In the high density limit, the differences between $G^{\infty }$ of LJ and WCA fluids seem to disappear. At high densities, the repulsive part of the interaction potential, which is the same for WCA and LJ, becomes more important. Hence, the two fluids are expected to behave in the same way as the density increases. This is shown in Figure 9b.

### 3.2. Estimation of Surface Effects

An important objective of this work is to investigate surface effects of finite systems used to compute KB integrals. As mentioned earlier, there are three possible approaches to compute the surface term in the thermodynamic limit $F^{\infty }$. Similar to the estimation of $G^{\infty }$, the surface term of the WCA fluid is computed from systems with varying number of particles *N* at the same thermodynamic state.

**Figure 10:**
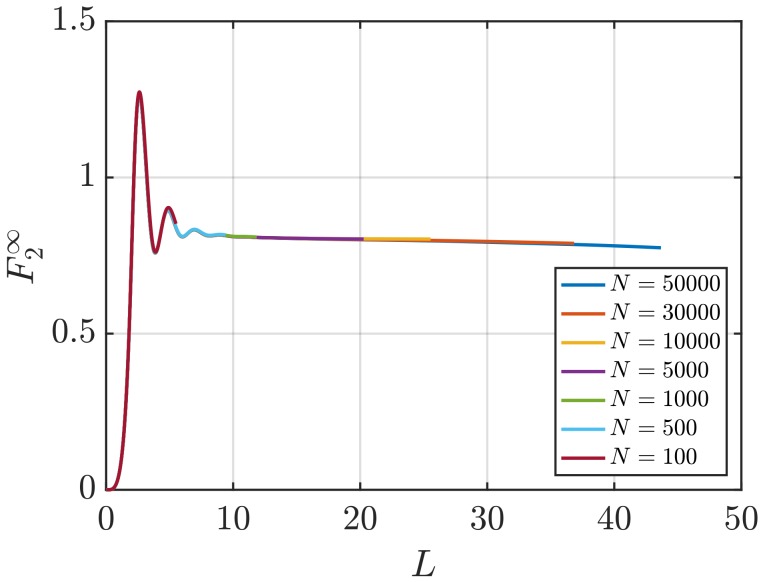
Estimation of the surface term in the thermodynamic limit $F_{2}^{\infty }$ (Equation (11)) vs. *L* for the WCA fluid at T=2 and ρ=0.6 (dimensionless units). The values of $F_{2}^{\infty }$ are computed for systems with varying number of molecules *N*. The used RDFs are provided in Figure 1.

**Figure 11:**
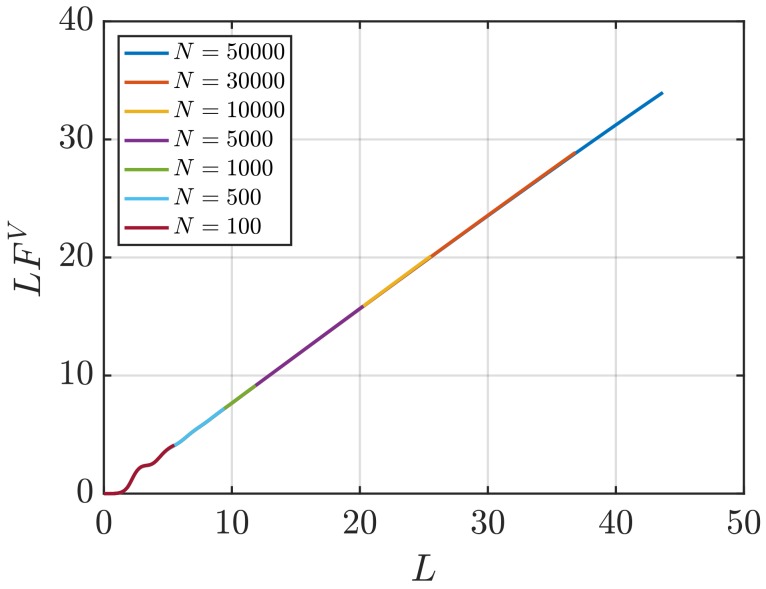
Surface effects of finite subvolumes multiplied by the diameter of the subvolume $LF^{v}$ (Equation (10)) vs. *L* for the WCA fluid at T=2 and ρ=0.6 (dimensionless units). The values of $G^{v}$ are computed for systems with varying number of molecules *N*. The used RDFs are provided in Figure 1.

In Figure 10, estimations of the surface term in the thermodynamic limit $F_{2}^{\infty }$ (Equation (11)) are presented as a function of *L*. Unlike the values of $G_{2}^{\infty }$, a plateau where the values can be averaged is not easily identified. Alternatively, it is possible to consider the scaling of the values of the surface term of finite subvolumes $F^{V}$ (Equation (10)). Figure 11 shows the scaling of $LF^{V}$ with *L* for the same WCA fluid. As in the case of the scaling of $LG^{V}$, a linear regime to be fitted is easily identified. The slope corresponds to the value of $F^{\infty }$. Additionally, the value of $F^{\infty }$ can be estimated from the intercept with the vertical axis of the line formed from the scaling of $LG^{V}$ with *L*. The latter two approaches require smaller system sizes than the direct estimation $F_{2}^{\infty }$. For instance, Figure 11 shows that systems with as few as N=1000 provide a clear linear range that can be used to estimate $F^{\infty }$. It is of interest to investigate whether the different available methods to find $F^{\infty }$ result in matching estimations. The values of $F^{\infty }$ computed using the three methods considered in this work are listed in Table 5. Results are shown for systems with varying number of particles. While acceptable statistics are achieved for most methods and system sizes, the values of $F^{\infty }$ from the three different methods agree less well than the corresponding values of $G^{\infty }$. This can be attributed to the larger statistical errors obtained when compared to estimating $G^{\infty }$.

**Figure 12:**
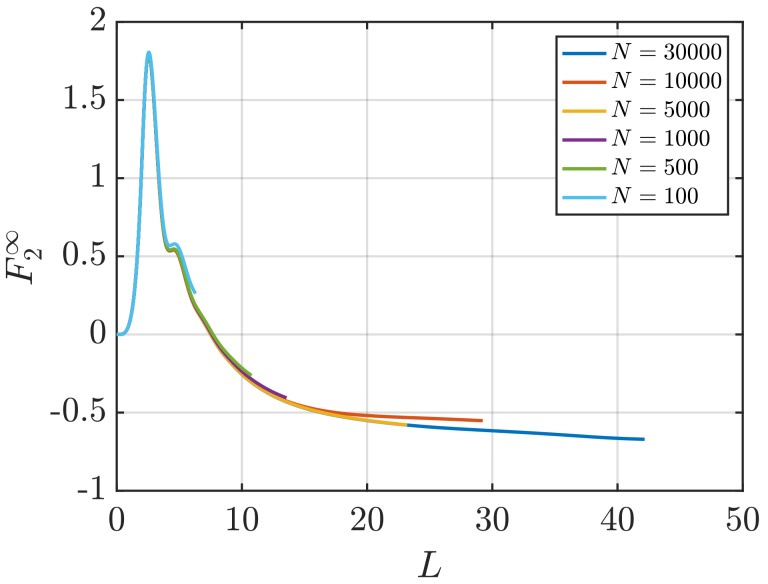
Estimation of the surface term in the thermodynamic limit $F_{2}^{\infty }$ (Equation (11)) vs. *L* for the LJ fluid at T=2 and ρ=0.4 (dimensionless units). The values of $F_{2}^{\infty }$ are computed for systems with varying number of molecules *N*. The used RDFs are provided in Figure 5.

**Figure 13:**
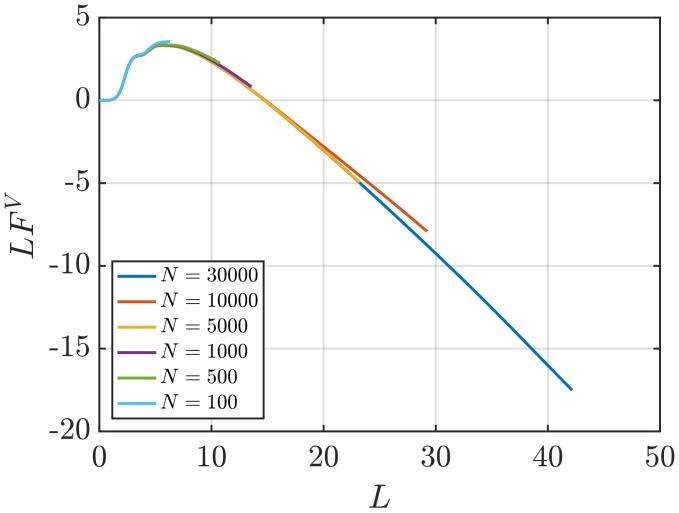
Surface effects of finite subvolumes multiplied by the diameter of the subvolume $LF^{V}$ (Equation (10)) vs. *L* for the LJ fluid at T=2 and ρ=0.4 (dimensionless units). The values of $G^{V}$ are computed for systems with varying number of molecules *N*. The used RDFs are provided in Figure 5.

As in the case of computing $G^{\infty }$, using scaling of $LG^{V}$ provides estimations of the surface term using systems smaller than those required by the other methods. This effect is more significant when looking at a system of LJ particles at a relatively low density. Table 6 provides the values of $F^{\infty }$ for a LJ fluid at ρ=0.4, computed using the methods studied in this work. In Figure 12 and Figure 13 the scaling of $F_{2}^{\infty }$ with *L* as well as the scaling of $LF^{V}$ with *L* are shown. These plots illustrate that linear regions are not easily identified to compute $F^{\infty }$, in contrast to the higher density case with ρ=0.6 (Figure 10 and Figure 11). As a result, when computing surface effects, the scaling of $LG^{V}$ with *L* is recommended.

#### Effect of System Size and Density

**Figure 14:**
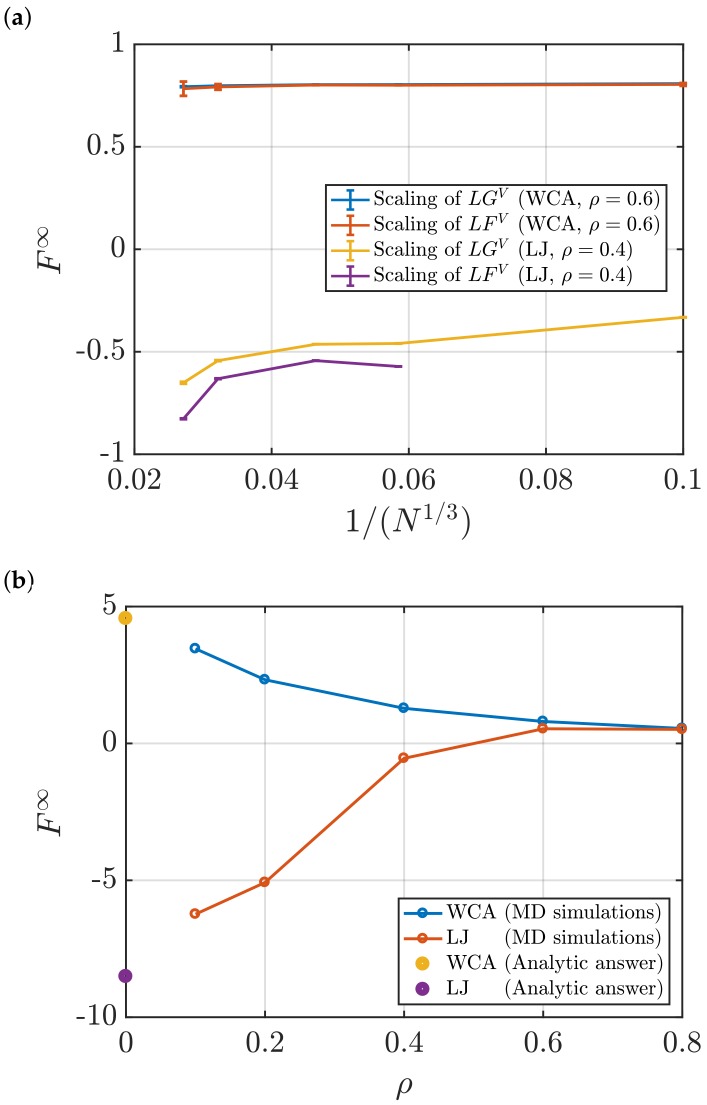
(**a**) Surface term in the thermodynamic limit *F*^∞^ as a function of the size of the system for the WCA fluid at *ρ* = 0.6, and the LJ fluid at *ρ* = 0.4. Both fluids are simulated at *T* = 2 (dimensionless units). (**b**) *F*^∞^ as a function of dimensionless density *ρ* of LJ and WCA systems at *T* = 2. For all densities, the same number of particles is used, *N* = 10,000.

Figure 14a shows the dependence of $F^{\infty }$ with inverse system size. Specifically, $F^{\infty }$ decreases as ${\left(1/N\right)}^{1/3}$. This is observed for surface terms computed using the scaling of $LF^{V}$ with *L* as well as values computed using the scaling of $LG^{V}$ with *L*. In Figure 14a, the error bars of the values of $F^{\infty }$ vary with *N* in a similar manner as the values of $G^{\infty }$. Statistical uncertainties of the systems studied in this work are provided in Table 5 and Table 6.

As for KB integrals, the surface terms $F^{\infty }$ can be determined accurately as a function of the density. Figure 14b shows the values of $F^{\infty }$ with density for LJ and WCA fluids. The surface term is estimated for the range ρ=0.1−0.8. At ρ→0, $F^{\infty }$ is computed analytically using Equation (10) and g(r)=exp[−βu(r)]. In the low density limit, the surface terms computed in this work approach the theoretical value. In the high density limit, differences between surface term of LJ and WCA disappear due to dominating repulsive interactions, which are the same for the LJ and WCA potentials. From Figure 9b and Figure 14b, a comparison between the values of $G^{\infty }$ and $F^{\infty }$ can be made. Both values change in the same manner with the density of the system. For all densities, surface terms $F^{\infty }$ seem to have the same order of magnitude as KB integrals $G^{\infty }$. This indicates the significant contribution of surface effects of finite systems used to compute KB integrals in the thermodynamic limit. The last observation applies to a LJ fluid as well as a WCA fluid.

## 4. Conclusions

In this work, KB integrals and surface effects in the thermodynamic limit were computed for systems of LJ and WCA fluids. Using MD simulations, RDFs of the LJ and WCA systems of different sizes were computed. Different methods were used to estimate KB integrals $G^{\infty }$ from RDFs of finite systems: scaling of $G^{V}$ with 1/L, direct estimation integrals $G_{2}$, and the scaling of $LG^{V}$ with *L*. The three methods were found to provide reliable estimates of $G^{\infty }$. Differences between the three methods mainly arise from the size of the system required to obtain an accurate estimation. The scaling of $LG^{V}$ with *L* was found to require smaller systems when compared to other methods. Moreover, the scaling of $LG^{V}$ was found the easiest to implement for estimating KB integrals and it provides a suitable estimate of surface effects. Estimating the surface term in the thermodynamic limit $F^{\infty }$ is possible from: the finite integral $F_{2}^{\infty }$, the scaling of $LF^{V}$ with *L*, as well as the scaling of $LG^{V}$ with *L*. For all methods, the surface term $F^{\infty }$ was found to decrease with increasing system size. The magnitude of the values of $F^{\infty }$ were found to be the same as the magnitude of the KB integrals $G^{\infty }$. Both quantities were found to change in the same manner with the density of the system. KB integrals and surface terms were computed for LJ and WCA fluids at different densities. The differences between KB integrals of the two systems, LJ and WCA, vanish for high densities as the structure is dominated by repulsive interactions.

## Figures and Tables

**Table 1:** A brief description of the methods used in this work to estimate Kirkwood–Buff (KB) integrals in the thermodynamic limit $G^{\infty }$ using radial distribution functions (RDFs) computed from finite systems.

Method	Equations	Description
1. Scaling of $G^{V}$ with 1/L	(4) and (5)	$G^{\infty }$ is obtained from extrapolating the linear regime of the scaling to 1/L→0.
2. Direct estimation $G_{2}$	(6) and (7)	A plateau in $G_{2}$ is identified when plotted as a function of *L*. To estimate $G^{\infty }$, values of $G_{2}$ in this plateau are averaged.
3. Scaling of $LG^{V}$ with *L*	(4) and (12)	To find $G^{\infty }$, the slope of the linear part of the scaling is computed.

**Table 2:** A brief description of the methods used in this work to estimate the surface term in the thermodynamic limit $F^{\infty }$ using RDFs computed from finite systems.

Method	Equations	Description
1. Direct estimation $F_{2}^{\infty }$	(11)	A plateau in $F_{2}^{\infty }$ is identified when plotted as a function of *L*. To estimate $F^{\infty }$, values of $F_{2}^{\infty }$ in this plateau are averaged.
2. Scaling of $LF^{V}$ with 1/L	(9) and (10)	To find $F^{\infty }$, the slope of the linear part of the scaling is computed.
3. Scaling of $LG^{V}$ with 1/L	(4) and (12)	To find $F^{\infty }$, the intercept of the linear part of the scaling is computed.

**Table 3:** KB integrals in the thermodynamic limit $G^{\infty }$ for a WCA system at T=2 and ρ=0.6 (dimensionless units). Values of $G^{\infty }$ are computed from systems with various number of particles *N* and using the different methods listed in Table 1.

*N*	Scaling of $G^{V}$ with 1/L	Direct Estimation $G_{2}$	Scaling of ${\mathrm{LG}}^{V}$ with *L*
500	−1.5063±0.0003	n/a	−1.5057±0.0008
1000	−1.5027±0.0000	n/a	−1.5028±0.0002
5000	−1.5012±0.0000	−1.5017±0.0004	−1.5013±0.0002
10,000	−1.5012±0.0000	−1.5015±0.0004	−1.5012±0.0001
30,000	−1.5004±0.0001	−1.5007±0.0007	−1.5003±0.0006
50,000	−1.4999±0.0001	−1.5002±0.0009	−1.500±0.001

**Table 4:** KB integrals in the thermodynamic limit $G^{\infty }$ for a LJ system at T=2 and ρ=0.4 (dimensionless units). Values of $G^{\infty }$ are computed from systems with various number of particles *N* and using the different methods listed in Table 1.

*N*	Scaling of $G^{V}$ with 1/L	Direct Estimation $G_{2}$	Scaling of ${\mathrm{LG}}^{V}$ with *L*
500	n/a	n/a	−1.1593±0.0001
1000	−1.1395±0.0001	n/a	−1.1390±0.0008
5000	−1.1161±0.0006	−1.13±0.02	−1.114±0.004
10,000	−1.1156±0.0005	−1.13±0.02	−1.114±0.004
30,000	−1.1064±0.0009	−1.12±0.02	−1.10±0.01

**Table 5:** Surface term in the thermodynamic limit $F^{\infty }$ for a WCA system at T=2 and ρ=0.6 (dimensionless units). Values of $F^{\infty }$ are computed from systems with various number of particles *N* and using the different methods listed in Table 2.

*N*	Direct Estimation $F_{2}^{\infty }$	Scaling of ${\mathrm{LG}}^{V}$ with *L*	Scaling of ${\mathrm{LF}}^{V}$ with *L*
500	n/a	0.8168±0.0008	n/a
1000	n/a	0.8082±0.0002	0.804±0.004
5000	0.801±0.002	0.8036±0.0002	0.8004±0.0002
10,000	0.8013±0.0004	0.8034±0.0001	0.8013±0.0003
30,000	0.795±0.005	0.7979±0.0006	0.79±0.01
50,000	0.79±0.01	0.793±0.001	0.78±0.02

**Table 6:** Surface term in the thermodynamic limit $F^{\infty }$ for a LJ system at T=2 and ρ=0.4 (dimensionless units). Values of $F^{\infty }$ are computed from systems with various number of particles *N* and using the different methods listed in Table 2.

*N*	Direct Estimation $F_{2}^{\infty }$	Scaling of ${\mathrm{LG}}^{V}$ with *L*	Scaling of ${\mathrm{LF}}^{V}$ with *L*
500	n/a	−0.2483±0.0001	n/a
1000	n/a	−0.3320±0.0008	n/a
5000	−0.53±0.04	−0.460±0.004	−0.5718±0.0001
10,000	−0.52±0.03	−0.464±0.004	−0.5433±0.0004
30,000	−0.60±0.06	−0.543±0.008	−0.6315±0.0009

## Supplementary Materials

The following are available at https://www.mdpi.com/2079-4991/10/4/771/s1, Tables S1–S16: KB integrals G${}^{\infty }$ and the surface term F${}^{\infty }$ in the thermodynamic limit are provided at different densities for the LJ and WCA fluids. For each interaction potential, G${}^{\infty }$ and F${}^{\infty }$ are computed at a dimensionless temperature *T* = 2 and the following dimensionless densities: ρ = 0.2, ρ = 0.4, ρ = 0.6, and ρ = 0.8.
