# Supplementary material for: Kirkwood-Buff Integrals Using Molecular Simulation: Estimation of Surface Effects
Source: Nanomaterials (Basel). 2020 Apr 16;10(4):771. doi: 10.3390/nano10040771 (PMC7221661; doi:10.3390/nano10040771)
Supplement: Supplementary file 1 [file nanomaterials-10-00771-s001.pdf]

Supporting Information
Kirkwood-Buff integrals using molecular simulation: estimation of surface effects

Noura Dawass[1], Peter Krüger[2], Sondre K. Schnell[3], Othonas A. Moultos[1], Ioannis G. Economou[4,5], Thijs J. H. Vlugt[1], Jean-Marc Simon*[6]

1 Engineering Thermodynamics, Process & Energy Department, Faculty of Mechanical, Maritime and Materials Engineering, Delft University of Technology, Leeghwaterstraat 39, 2628CB Delft, The Netherlands
2 Graduate School of Advanced Integration Science, Chiba University, Chiba 263-8522, Japan
3 Department of Materials Science and Engineering, NTNU, N-7491, Trondheim, Norway
4 National Center for Scientific Research "Demokritos", Institute of Nanoscience and Nanotechnology, Molecular Thermodynamics and Modelling of Materials Laboratory, GR – 153 10, Aghia Paraskevi Attikis, Greece
5 Texas A&M University at Qatar, Chemical Engineering Program, Education City, PO Box 23874, Doha, Qatar
6 ICB, UMR 6303 CNRS - Université de Bourgogne, F-21078, Dijon, France
* Correspondence: jmsimon@u-bourgogne.fr

Version March 13, 2020 submitted to Nanomaterials

In this Supporting Information, KB integrals G^∞ and the surface term F^∞ in the thermodynamic limit are provided at different densities for the LJ and WCA fluids. For each interaction potential, G^∞ and F^∞ are computed at a dimensionless temperature $T = 2$ and the following dimensionless densities: $\rho = 0.2$, $\rho = 0.4$, $\rho = 0.6$, and $\rho = 0.8$. Values obtained using different estimation methods are discussed in the main text.

Table S1. KB integrals in the thermodynamic limit G^{∞} for a LJ system at $T = 2$ and $\rho = 0.2$ (dimensionless units). Values of G^{∞} are computed from systems with various number of particles N and using the different methods studied in this work.

N	Scaling of G^V with $1/L$	Direct estimation G_2	Scaling of LG^V with L
5000	0.219 ± 0.003	n/a	0.23 ± 0.02
10000	0.244 ± 0.004	0.20 ± 0.09	0.26 ± 0.04
30000	0.204 ± 0.002	0.18 ± 0.06	0.21 ± 0.01

Table S2. Surface term in the thermodynamic limit F^{∞} for a LJ system at $T = 2$ and $\rho = 0.2$ (dimensionless units). Values of F^{∞} are computed from systems with various number of particles N and using the different methods studied in this work.

N	Direct estimation F_2^{∞}	Scaling of LG^V with L	Scaling of LF^V with L
5000	n/a	-4.39 ± 0.02	n/a
10000	-4.8 ± 0.4	-4.63 ± 0.04	-5.1 ± 0.3
30000	-4.4 ± 0.1	-4.27 ± 0.01	-4.4 ± 0.2

Table S3. KB integrals in the thermodynamic limit G^{∞} for a LJ system at $T = 2$ and $\rho = 0.4$ (dimensionless units). Values of G^{∞} are computed from systems with various number of particles N and using the different methods studied in this work.

N	Scaling of G^V with $1/L$	Direct estimation G_2	Scaling of LG^V with L
500	n/a	n/a	-1.1593 ± 0.0001
1000	-1.1395 ± 0.0001	n/a	-1.1390 ± 0.0008
5000	-1.1161 ± 0.0006	-1.13 ± 0.02	-1.114 ± 0.004
10000	-1.1156 ± 0.0005	-1.13 ± 0.02	-1.114 ± 0.004
30000	-1.1064 ± 0.0009	-1.12 ± 0.02	-1.10 ± 0.01

Table S4. Surface term in the thermodynamic limit F^{∞} for a LJ system at $T = 2$ and $\rho = 0.4$ (dimensionless units). Values of F^{∞} are computed from systems with various number of particles N and using the different methods studied in this work.

N	Direct estimation F_2^{∞}	Scaling of LG^V with L	Scaling of LF^V with L
500	n/a	-0.2483 ± 0.0001	n/a
1000	n/a	-0.3320 ± 0.0008	n/a
5000	-0.53 ± 0.04	-0.460 ± 0.004	-0.5718 ± 0.0001
10000	-0.52 ± 0.03	-0.464 ± 0.004	-0.5433 ± 0.0004
30000	-0.60 ± 0.06	-0.543 ± 0.008	-0.6315 ± 0.0009

Table S5. KB integrals in the thermodynamic limit G^{∞} for a LJ system at $T = 2$ and $\rho = 0.6$ (dimensionless units). Values of G^{∞} are computed from systems with various number of particles N and using the different methods studied in this work.

N	Scaling of G^V with $1/L$	Direct estimation G_2	Scaling of LG^V with L
500	-1.3623 ± 0.0007	n/a	-1.363 ± 0.002
1000	-1.3558 ± 0.0001	n/a	-1.3556 ± 0.0004
5000	-1.3507 ± 0.0000	-1.3519 ± 0.0006	-1.3507 ± 0.0000
10000	-1.3488 ± 0.0000	-1.350 ± 0.001	-1.3487 ± 0.0003
30000	-1.3514 ± 0.0000	-1.3518 ± 0.0005	-1.3514 ± 0.0002
50000	-1.3471 ± 0.0001	-1.3480 ± 0.002	-1.347 ± 0.001

Table S6. Surface term in the thermodynamic limit F^{∞} for a LJ system at $T = 2$ and $\rho = 0.6$ (dimensionless units). Values of F^{∞} are computed from systems with various number of particles N and using the different methods studied in this work.

N	Direct estimation F_2^{∞}	Scaling of LG^V with L	Scaling of LF^V with L
500	n/a	0.604 ± 0.002	n/a
1000	n/a	0.5792 ± 0.0004	n/a
5000	0.5512 ± 0.0005	0.5567 ± 0.0000	0.5494 ± 0.0001
10000	0.540 ± 0.004	0.5444 ± 0.0003	0.535 ± 0.002
30000	0.557 ± 0.002	0.5609 ± 0.0002	0.557 ± 0.004
50000	0.53 ± 0.01	0.529 ± 0.001	0.52 ± 0.02

Table S7. KB integrals in the thermodynamic limit G^{∞} for a LJ system at $T = 2$ and $\rho = 0.8$ (dimensionless units). Values of G^{∞} are computed from systems with various number of particles N and using the different methods studied in this work.

N	Scaling of G^V with $1/L$	Direct estimation G_2	Scaling of LG^V with L
500	-1.178 ± 0.001	n/a	-1.178 ± 0.004
1000	-1.1687 ± 0.0002	n/a	-1.169 ± 0.001
5000	-1.1695 ± 0.0001	-1.1702 ± 0.0008	-1.1695 ± 0.0006
10000	-1.1695 ± 0.0001	-1.1700 ± 0.0007	-1.1695 ± 0.0005
30000	-1.1694 ± 0.0001	-1.1697 ± 0.0006	-1.1694 ± 0.0004
50000	-1.1691 ± 0.0001	-1.1693 ± 0.0007	-1.1690 ± 0.0004

Table S8. Surface term in the thermodynamic limit F^{∞} for a LJ system at $T = 2$ and $\rho = 0.8$ (dimensionless units). Values of F^{∞} are computed from systems with various number of particles N and using the different methods studied in this work.

N	Direct estimation F_2^{∞}	Scaling of LG^V with L	Scaling of LF^V with L
500	n/a	0.545 ± 0.004	n/a
1000	n/a	0.512 ± 0.001	n/a
5000	0.514 ± 0.004	0.5152 ± 0.0006	0.512 ± 0.002
10000	0.514 ± 0.001	0.5154 ± 0.0005	0.513 ± 0.002
30000	0.513 ± 0.001	0.5143 ± 0.0004	0.512 ± 0.002
50000	0.510 ± 0.002	0.5119 ± 0.0004	0.509 ± 0.002

Table S9. KB integrals in the thermodynamic limit G^{∞} for a WCA system at $T = 2$ and $\rho = 0.2$ (dimensionless units). Values of G^{∞} are computed from systems with various number of particles N and using the different method studied in this work.

N	Scaling of G^V with $1/L$	Direct estimation G_2	Scaling of LG^V with L
500	-2.7413 ± 0.0000	n/a	-2.7413 ± 0.0000
1000	-2.7396 ± 0.0000	n/a	-2.7396 ± 0.0000
5000	-2.7369 ± 0.0000	-2.7372 ± 0.0004	-2.7367 ± 0.0004
10000	-2.7428 ± 0.0001	-2.742 ± 0.002	-2.743 ± 0.001
30000	-2.7419 ± 0.0005	-2.741 ± 0.005	-2.74 ± 0.01
50000	-2.7298 ± 0.0009	-2.731 ± 0.008	-2.73 ± 0.02

Table S10. Surface term in the thermodynamic limit F^{∞} for a WCA system at $T = 2$ and $\rho = 0.2$ (dimensionless units). Values of F^{∞} are computed from systems with various number of particles N and using the different methods studied in this work.

N	Direct estimation F_2^{∞}	Scaling of LG^V with L	Scaling of LF^V with L
500	n/a	2.2674 ± 0.0000	2.2752 ± 0.0001
1000	n/a	2.2659 ± 0.0000	2.2699 ± 0.0001
5000	2.26 ± 0.01	2.2499 ± 0.0004	2.246 ± 0.007
10000	2.31 ± 0.02	2.308 ± 0.001	2.33 ± 0.02
30000	2.35 ± 0.02	2.34 ± 0.01	2.40 ± 0.27
50000	2.08 ± 0.09	2.10 ± 0.02	1.98 ± 0.45

Table S11. KB integrals in the thermodynamic limit G^{∞} for a WCA system at $T = 2$ and $\rho = 0.4$ (dimensionless units). Values of G^{∞} are computed from systems with various number of particles N and using the different methods studied in this work.

N	Scaling of G^V with $1/L$	Direct estimation G_2	Scaling of LG^V with L
500	-1.9776 ± 0.0000	n/a	-1.9774 ± 0.0002
1000	-1.9748 ± 0.0000	-1.977 ± 0.002	-1.9748 ± 0.0000
5000	-1.9737 ± 0.0000	-1.9740 ± 0.0003	-1.9737 ± 0.0000
10000	-1.9738 ± 0.0000	-1.9739 ± 0.0002	-1.9738 ± 0.0002
30000	-1.9766 ± 0.0001	-1.976 ± 0.001	-1.977 ± 0.001
50000	-1.9731 ± 0.0001	-1.9732 ± 0.0008	-1.973 ± 0.002

Table S12. Surface term in the thermodynamic limit F^{∞} for a WCA system at $T = 2$ and $\rho = 0.4$ (dimensionless units). Values of F^{∞} are computed from systems with various number of particles N and using the different methods studied in this work.

N	Direct estimation F_2^{∞}	Scaling of LG^V with L	Scaling of LF^V with L
500	n/a	1.2895 ± 0.0002	1.2843 ± 0.0008
1000	n/a	1.2810 ± 0.0000	1.2810 ± 0.0009
5000	1.281 ± 0.005	1.2792 ± 0.0000	1.2774 ± 0.0001
10000	1.282 ± 0.004	1.2815 ± 0.0002	1.282 ± 0.003
30000	1.30 ± 0.02	1.305 ± 0.001	1.32 ± 0.02
50000	1.27 ± 0.02	1.270 ± 0.002	1.26 ± 0.03

Table S13. KB integrals in the thermodynamic limit G^{∞} for a WCA system at $T = 2$ and $\rho = 0.6$ (dimensionless units). Values of G^{∞} are computed from systems with various number of particles N and using the different methods studied in this work.

N	Scaling of G^V with $1/L$	Direct estimation G_2	Scaling of LG^V with L
500	-1.5063 ± 0.0003	n/a	-1.5057 ± 0.0008
1000	-1.5027 ± 0.0000	n/a	-1.5028 ± 0.0002
5000	-1.5012 ± 0.0000	-1.5017 ± 0.0004	-1.5013 ± 0.0002
10000	-1.5012 ± 0.0000	-1.5015 ± 0.0004	-1.5012 ± 0.0001
30000	-1.5004 ± 0.0001	-1.5007 ± 0.0007	-1.5003 ± 0.0006
50000	-1.4999 ± 0.0001	-1.5002 ± 0.0009	-1.500 ± 0.001

Table S14. Surface term in the thermodynamic limit F^{∞} for a WCA system at $T = 2$ and $\rho = 0.6$ (dimensionless units). Values of F^{∞} are computed from systems with various number of particles N and using the different methods studied in this work.

N	Direct estimation F_2^{∞}	Scaling of LG^V with L	Scaling of LF^V with L
500	n/a	0.8168 ± 0.0008	n/a
1000	n/a	0.8082 ± 0.0002	0.804 ± 0.004
5000	0.801 ± 0.002	0.8036 ± 0.0002	0.8004 ± 0.0002
10000	0.8013 ± 0.0004	0.8034 ± 0.0001	0.8013 ± 0.0003
30000	0.795 ± 0.005	0.7979 ± 0.0006	0.79 ± 0.01
50000	0.79 ± 0.01	0.793 ± 0.001	0.78 ± 0.02

Table S15. KB integrals in the thermodynamic limit G^{∞} for a WCA system at $T = 2$ and $\rho = 0.8$ (dimensionless units). Values of G^{∞} are computed from systems with various number of particles N and using the different methods studied in this work.

N	Scaling of G^V with $1/L$	Direct estimation G_2	Scaling of LG^V with L
500	n/a	n/a	-1.189 ± 0.002
1000	-1.1937 ± 0.0000	n/a	-1.1908 ± 0.0008
5000	-1.1909 ± 0.0000	-1.1914 ± 0.0005	-1.1909 ± 0.0004
10000	-1.1909 ± 0.0000	-1.1913 ± 0.0005	-1.1901 ± 0.0003
30000	-1.1908 ± 0.0000	-1.1910 ± 0.0004	-1.1908 ± 0.0003
50000	-1.1905 ± 0.0000	-1.1907 ± 0.0005	-1.1904 ± 0.0004

Table S16. Surface term in the thermodynamic limit F^{∞} for a WCA system at $T = 2$ and $\rho = 0.8$ (dimensionless units). Values of F^{∞} are computed from systems with various number of particles N and using the different methods studied in this work.

N	Direct estimation F_2^{∞}	Scaling of LG^V with L	Scaling of LF^V with L
500	n/a	0.538 ± 0.002	n/a
1000	n/a	0.5456 ± 0.0008	n/a
5000	0.55 ± 0.01	0.5480 ± 0.0004	0.546 ± 0.001
10000	0.548 ± 0.005	0.5483 ± 0.0003	0.547 ± 0.001
30000	0.547 ± 0.002	0.5477 ± 0.0003	0.547 ± 0.001
50000	0.543 ± 0.003	0.5449 ± 0.0004	0.542 ± 0.004

© 2020 by the authors. Submitted to *Nanomaterials* for possible open access publication under the terms and conditions of the Creative Commons Attribution (CC BY) license (http://creativecommons.org/licenses/by/4.0/).
